# Combining deep mutational scanning to heatmap of HLA class II binding of immunogenic sequences to preserve functionality and mitigate predicted immunogenicity

**DOI:** 10.3389/fimmu.2023.1197919

**Published:** 2023-07-28

**Authors:** Coline Sivelle, Raphael Sierocki, Youen Lesparre, Aurore Lomet, Wagner Quintilio, Steven Dubois, Evelyne Correia, Ana Maria Moro, Bernard Maillère, Hervé Nozach

**Affiliations:** ^1^ Université de Paris-Saclay, CEA, INRAE, Département Médicaments et Technologies pour la Santé, SIMoS, Gif-sur-Yvette, France; ^2^ Deeptope SAS, Orsay, France; ^3^ CEA List, Université Paris-Saclay, Palaiseau, France; ^4^ Biopharmaceuticals Laboratory, Butantan Institute, Sao Paulo, Brazil

**Keywords:** immunogenicity, T-cell epitope, yeast surface display, antibody engineering, deep mutational scanning

## Abstract

Removal of CD4 T cell epitopes from therapeutic antibody sequences is expected to mitigate their potential immunogenicity, but its application is complicated by the location of their T cell epitopes, which mainly overlap with complementarity-determining regions. We therefore evaluated the flexibility of antibody sequences to reduce the predicted affinity of corresponding peptides for HLA II molecules and to maintain antibody binding to its target in order to guide antibody engineering for mitigation of predicted immunogenicity. Permissive substitutions to reduce affinity of peptides for HLA II molecules were identified by establishing a heatmap of HLA class II binding using T-cell epitope prediction tools, while permissive substitutions preserving binding to the target were identified by means of deep mutational scanning and yeast surface display. Combinatorial libraries were then designed to identify active clones. Applied to adalimumab, an anti-TNFα human antibody, this approach identified 200 mutants with a lower HLA binding score than adalimumab. Three mutants were produced as full-length antibodies and showed a higher affinity for TNFα and neutralization ability than adalimumab. This study also sheds light on the permissiveness of antibody sequences with regard to functionality and predicted T cell epitope content.

## Introduction

Refinement of antibody display technologies has enabled the discovery of a large array of antibodies with therapeutic potential (1). The key feature of display technologies is the linkage of displayed antibody variants to their genetic information, allowing isolation of rare active variants and their identification by sequencing of their DNA. From these common principles, specific procedures have been developed not only to sort active mutants but also to improve their attributes by engineering their sequence. One of the main applications is affinity maturation, which can be achieved by using combinatorial libraries (2), Complementarity-determining region (CDR)-targeted mutagenesis (3, 4) or light chain shuffling (5, 6). Enlargement of antibody selectivity to neutralize emerging viral variants has also been recently achieved using Deep Mutational Scanning (DMS) (7). DMS assesses the impact of every possible single amino acid substitution in a selected protein on the interaction with another one (8, 9). DMS reveals therefore key positions of the interaction in an antibody epitope or paratope and permissive substitutions, which either preserve or enhance antibody binding. These substitutions were advantageously introduced into combinatorial libraries to leverage the individual and synergistic impact of the most active positions (7). Specificity and affinity of antibodies can therefore be finely tuned by combining display technologies and large mutational strategies.

However, therapeutic antibodies may be immunogenic and might elicit in the treated patients the production of anti-drug antibodies (ADA), which can alter the pharmacokinetics of the therapeutic antibodies, diminish their clinical efficacy or induce hypersensitivity reactions (10–12). Immunogenicity is variable from one antibody to another as its incidence depends on its sequence and in particular on its content of CD4 T cell epitopes (13–16). CD4 T cell epitopes are peptides, which result from the proteolytic degradation of antigens in the endosomes of dendritic cells and are displayed by HLA class II molecules at the surface of the cells for recognition by CD4 T lymphocytes. Activation of CD4 T lymphocytes provides the help required by B lymphocytes to produce ADA and explains the requirement for T cell epitopes in inducing ADA production. In contrast to the binding properties of the antibody, immunogenicity cannot be directly assessed during the early stages of antibody development, including the phase of candidate selection, and is only evaluated during the clinical phases. Because of the key role of CD4 T cell epitopes in the triggering of the ADA response, early evaluation of the risk of immunogenicity of antibody candidates mainly relies on the search for these immunogenic sequences using appropriate software. Many software packages identify sequences with specific motifs of binding to HLA class II molecules and have been developed on the basis of different principles including sequence alignment, binding matrices and neuronal networks (17, 18). These programs are used to rank candidate antibodies based on the number of CD4 T cell epitopes. In addition, antibody sequences can be modified to eliminate predicted T cell epitopes with the potential to bind to HLA class II molecules, stimulate CD4 T cells and induce ADA production (19–21 14, 22). However, almost all CD4 T epitopes identified in human and humanized antibodies are located in the mutated regions of the variable antibody domains with respect to the germline sequence. These regions largely overlap with antibody CDRs, which are the peptide loops interacting with the antibody target (13–16). As a consequence, the modification of sequences to get rid of immunogenic sequences may also affect the function of the antibody and should therefore be designed so as to reduce immunogenicity and maintain biological function.

We therefore developed a method of de-immunization guided by the identification of substitutions, which reduce the binding of corresponding peptides to HLA class II molecules but preserve the capacity of the antibody to bind to its target. This identification is carried out by establishing a heatmap of interaction of the peptides with the HLA II molecules using prediction software and a heatmap of interaction with the antigen provided by DMS applied by the yeast display system. Selection of combinatorial libraries including identified substitutions leads to the selection of optimized molecules. This approach was applied to adalimumab, a human anti-human TNFα antibody, selected from bacterial phages expressing a library of variable human Ig fragments (23). Adalimumab is prescribed for the treatment of inflammatory diseases such as rheumatoid arthritis, ankylosing spondylitis and Crohn’s disease (24). Adalimumab elicits ADA responses in approximately one-third of patients, who often become resistant to the treatment (25).

## Materials and methods

### *In silico* prediction of HLA class II binding

HLA class II binding prediction was performed using the netMHCIIpan3.2 algorithm (17) to identify 9-mer sequences putatively interacting with HLA class II molecules (also called HLA binding cores). Human 9-mer germline sequences were excluded from the analysis (26) and the algorithm was applied to the two regions containing CD4 T cell epitopes (15), which encompassed the HCDR2 and HCDR3 regions. A panel of 46 alleles of HLA class II was selected to maximize the worldwide population coverage (27). This panel includes the HLA-DR DQ and DP allelic variants, which provide worldwide population (phenotypic) coverage of almost 90% at each locus and account for over 66% of all genes at each locus. For each allele and each 9-mer sequence, a percentile rank was calculated by comparing the value of each 9-mer sequence against the value of five million random peptides selected from the SWISSPROT database. A 9-mer sequence was considered as an HLA binding core for a percentile rank below 20%. The binding score of each 9-mer sequence and of each of the HCDR2 and HCDR3 peptide regions was defined as the number of predicted cores across the 46 HLA class II alleles. HLA class II binding prediction was also performed for sequences containing a single amino acid substitution for all positions considered. Differences in the number of predicted cores between the original sequence of each of the HCDR2 and HCDR3 peptide regions and the monosubstituted sequences were calculated and used to provide a heatmap of HLA class II binding.

### Yeast display vector construction

DNA cloning was performed with the SLiCE recombination cloning method (28) or the classic restriction/ligature method using *E. coli* DH5α strain (Invitrogen). The plasmid pSWAG adalimumab includes the DNA sequences coding for the VH and VL sequences of adalimumab. This plasmid is used to express adalimumab Fab by yeast surface display and is derived from previously described pCT-L7.5.1 with a galactose inducible bidirectional promoter GAL1/GAL10. The GAL10 promoter is used to express the light chain of the adalimumab Fab, whose gene is cloned between *NheI*/*SalI* restriction sites. The GAL1 promoter is used to express the heavy chain of the adalimumab Fab, whose gene is cloned between *NcoI*/*Pfl23II* restriction sites, followed by the Aga2p anchor sequence. Plasmid pCT-L7.5.1 was a gift from Prof. K. Dane Wittrup (Addgene plasmid # 42900).

### Yeast transformation and expression conditions

Preparation of competent yeast cells EBY100 (ATCC® MYA- 4941™) was performed according to Benatuil et al. (29). Then, EBY100 electrocompetent cells (400 µL) were mixed with 2-3 µg linearized plasmid DNA and 4-6 µg of VH DNA insert, and transferred to a pre-chilled electroporation cuvette (Bio-Rad, 165–2086) and pulsed at 2.5 kV, 25 mF (Bio-Rad Gene Pulser Xcell). Yeast cultures were performed in Erlenmeyer flasks at 30°C and 180 rpm in SD-CAA medium (6.7 g/L yeast nitrogen base without casamino acids, 20 g/L glucose, 5 g/L casamino acids, 100 mM sodium phosphate, pH 6.0). After passage to an OD_600_ of 0.25, cells were grown at 30°C until OD_600_ 0.5-1 and re-suspended in SG-CAA galactose induction medium (6.7 g/L yeast nitrogen base without casamino acids, 20 g/L galactose, 5 g/L casamino acids, 100 mM sodium phosphate, pH 6.0) and induced for 16–36 h at 20°C, 180 rpm.

### Generation and sorting of DMS and combinatorial libraries

Two DMS libraries with mutations corresponding to a single amino acid change were generated separately for the two CDRs (positions 46 to 64 and 89 to 104) by splicing by overlap extension PCR (SOE-PCR) using degenerate NNK primers. Libraries were cloned by recombination of VH genes in the digested adalimumab pSWAG plasmid using *NcoI*/*Pfl23II* restriction enzymes.

For each of the two DMS libraries, the cells to be sorted (3*10^7 cells) were washed with PBSF [phosphate-buffered saline (PBS), bovine serum albumin (BSA) 0.1%] buffer and incubated with 80 pM human biotinylated TNFα (ACROBiosystems) in 150 mL of PBSF at 20°C, 180 rpm for 3 hours. Cells were washed with ice-cold PBSF before incubation with PE-conjugated streptavidin (Thermo Fisher Scientific; Ref S866; 1:100 dilution) and Mouse anti-Human Kappa Light Chain Antibody APC conjugate (Thermo Fisher Scientific; catalog number MH10515; 1:100 dilution) and sorted with a BD FACS Aria™ III cytometer following the optimized sorting conditions described by Kowalsky et al. (30). For each sorting step, the number of labeled cells was greater than 50 times the expected diversity of the corresponding library.

Because of its large diversity, the HCDR3 library expressed by YSD was first screened by MACS as described by Chao et al. (31). In short, cells were incubated for 3 h at 20°C with 10 nM biotinylated TNFα in 1 mL of PBSF buffer. After two washes, Miltenyi anti-biotin microbeads (200 µL) were added and a 10-min incubation was performed. The cell suspension was loaded on a Miltenyi LS column placed on a magnet. After two passages through the column and two washes with 3 mL of buffer, the retained cells were cultured in SD-CAA medium.

For FACS, equilibrium selection was performed by sorting the top 2 to 5% of cells with the highest fluorescence using diagonal gates with decreasing concentrations of human biotinylated TNFα (3 nM, 1 nM or 500 pM, respectively). The final selection based on k_off_ values was performed by saturating the cells with 20 nM human biotinylated TNFα for 3 h at 20°C. After washing, an excess of 1 µM human non-biotinylated TNFα was added and incubated for another 24 h at 20°C. Again, the 2 to 5% of the cells showing the maximum remaining fluorescence were sorted with diagonal gates.

### Next-generation sequencing and data analysis

DNA plasmids were extracted using Zymoprep Yeast Plasmid Miniprep II (Zymo Research). Fragments corresponding to mutated areas were amplified, and Illumina® adaptors, sequencing primers and barcodes were added by 2 PCR reactions as described by Kowalsky et al. (30). Libraries were sequenced in paired-end sequencing using a Miseq (V2 2x150 bp) or an iSeq (2x150 bp). Each DMS library was sequenced with a 50X depth. Raw data were processed using the Galaxy environment (https://usegalaxy.org) with functions described by Blankenberg et al. (32). Briefly, sequences were paired (fastq-join) and filtered on the basis of quality score (above 30) (Filter FASTQ), and aligned on the native sequence (Align.seqs). The region of interest was extracted (Chop.seqs) and DNA sequences were translated (transeq) and grouped based on sequence (Group). The diversity of mutations was represented as weblogo (33) using samples of 1000 sequences. Statistical analysis performed using R included sequences for which there were at least 5 and 50 reads in the sequencing following the selection for DMS libraries and combinatorial libraries, respectively. Enrichment of a sequence is defined as follows:


$$\;\;\;Enrichment=\frac{F_{out}^{i}}{F_{in}^{i}}$$

Where $F_{out}^{i}$ is the final frequency of the sequence *i* in the library population after selection and $F_{in}^{i}$ is the initial frequency. For DMS libraries, mutants are represented by a relative binding score normalized by native sequence enrichment defined as follows:


$$\text{relative binding score}=log_{2}(\frac{\text{Enrichment}}{F_{out}^{wt}/F_{in}^{wt}})$$

Where $F_{out}^{wt}$ is the final frequency of the native sequence in the library population after selection and $F_{in}^{wt}$ is the initial frequency.

### Production and purification of Fab and IgG

The genes encoding the heavy and light chains of adalimumab and mutants were cloned into AbVec2.0-IGHG1 and AbVec1.1-IGKC, respectively (34). For Fab expression, the DNA sequence of the hinge and CH2 and CH3 regions were deleted and a DNA sequence encoding a 6xHis tag was added to the DNA sequence corresponding to the C-terminal end. Expression was performed via transient transfection of HEK293 Freestyle™ cells (HEK 293FS) as previously described by Subedi et al. (35). Seven days after transfection, the supernatant was purified on an AKTA purifier (GE Healthcare) using a 1 mL HisTrap Excel™ column for Fab and a 5 mL HiTrap™ Protein A HP column (GE Healthcare) according to the manufacturer’s recommendations. For IgG purification, a second purification step by size exclusion chromatography was performed using a HiPrep Sephacryl S200 (GE Healthcare) column. 5 mL of each sample was loaded on the column using a flow rate of 0.7 mL/min and PBS as running buffer.

### Affinity measurement by BioLayer Interferometry

Binding affinities were determined using an Octet RED96 instrument (Pall ForteBio). Biotinylated TNFα was loaded onto streptavidin biosensors (ForteBio) at a concentration of 20 nM for 2 min in PBS and Fab were then associated at different concentrations ranging from 0.625 to 15 nM. Association and dissociation steps were monitored for 20 min and 30 min, respectively. A global 1:1 Langmuir model was applied to obtain the affinity parameters, using the Data Analysis HT software provided with the Octet RED96 instrument.

### Neutralization of the TNFα-induced cytotoxicity assay

Neutralization of the TNFα -induced cytotoxicity assay was performed as described by Luchese et al. (36). A cell growth curve was established for murine L929 fibroblasts in DMEM/F12 (GE Healthcare) medium supplemented with 10% fetal bovine serum (Cultilab) and 2 mM glutamine. Antibodies were serially diluted from 250 ng/mL to 0.24 ng/mL (dilution factor 2) and incubated with 10 ng/mL TNFα for 30 min at room temperature prior to the addition of the mixture to the cells and actinomycin-D. Cell viability was quantified by the tetrazolium dye (MTT) method with 20% SDS solubilization at 37°C for 20 h. EC_50_ (half maximal effective concentration) was determined by GraphPad Prism 5® considering the results of three independent experiments and using as control L929 cells alone (negative control).

## Results

### Multiple substitutions in CD4 T cell epitopes of adalimumab are expected to reduce their binding to HLA class II molecules

**Figure 1:**
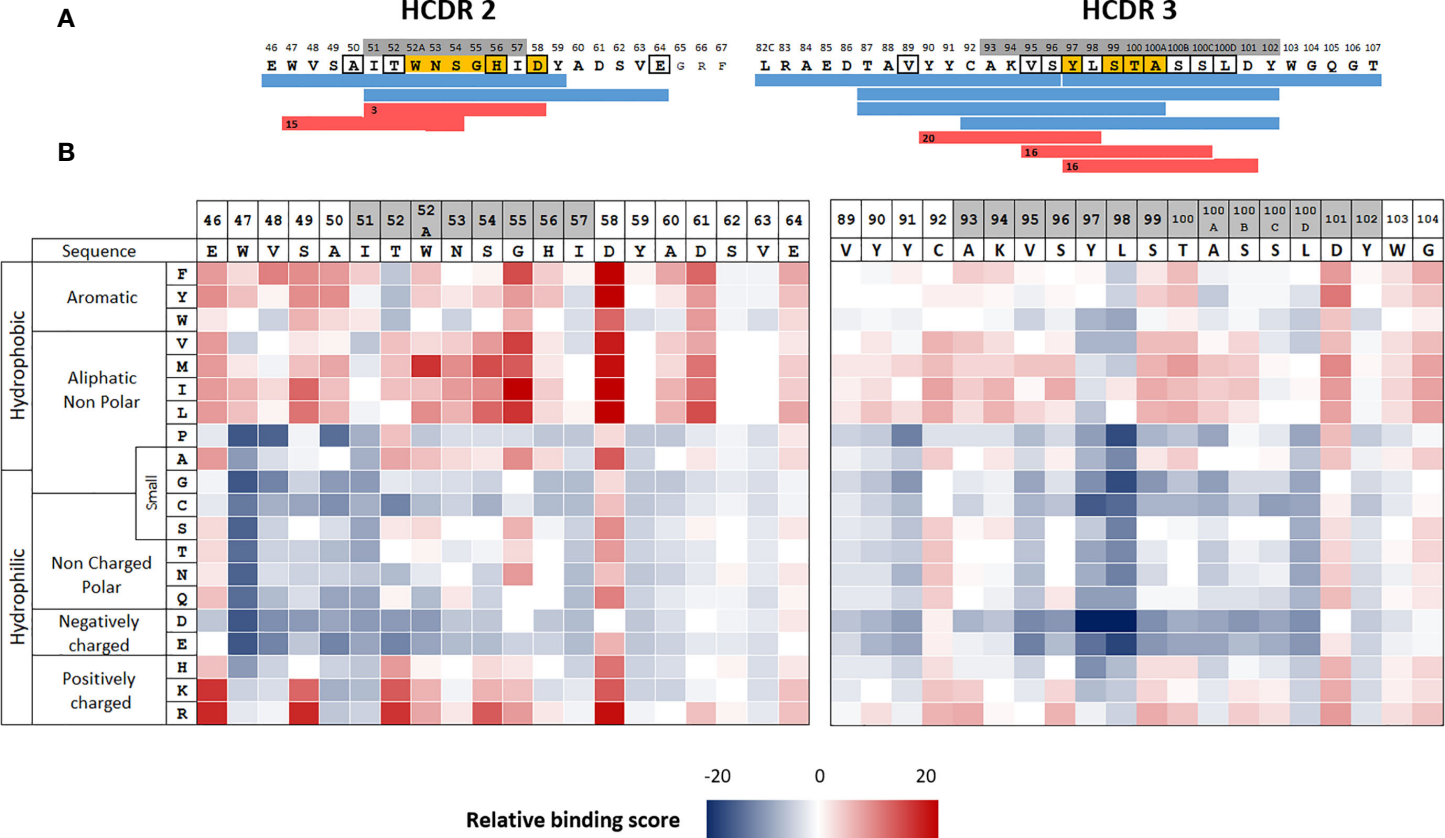
Location of the CD4 T-cell epitopes in the HCDR2 and HCDR3 regions of adalimumab and HLA II binding prediction. **(A)** CD4 T cell epitopes of adalimumab (blue) were identified mainly in CDR2 and CDR3 by generation of adalimumab-specific T cell lines (15). Binding cores of the T cell epitopes were predicted using netMHCIIpan3.2 (red). Amino acids framed in black correspond to mutations with respect to the best-fitting germline sequence (IGHV3-9*01,IGHJ4*02, IGHD2-2*0) retrieved from IMGT (37). CDRs are shown in grey. **(B)** heatmap of HLA binding prediction. HLA binding scores correspond to the number of binding cores identified by netMHCIIpan3.2 for the 46 selected HLA class II alleles. Binding to HLA class II molecules is considered as effective below the percentile threshold of 20%. These scores were calculated for all the monosubstituted sequence with the 20 amino acids at each position of the two adalimumab regions. Data are reported as increase (red) or decrease (blue) in binding score represented relative to the adalimumab sequence. Amino acid positions are numbered according to Kabat et al. which explains the presence of letters behind the numbers of some positions (38).

We selected adalimumab for this mutational study, as we had already identified its CD4 T cell epitopes in a previous study (15). T-cell epitopes of adalimumab were frequently found between residues L82c and T107, encompassing the HCDR3 and to a lesser degree between residues E46 and E64, encompassing HCDR2 (**Figure 1A**) (15). To predict interaction modalities of the T-cell epitopes with HLA class II molecules, we used the NetMHCIIpan 3.2 algorithm (17), by excluding human 9-mer sequences (26). The core of a CD4 T cell epitope is a sequence of 9 amino acids, embedded in the HLA class II binding groove and limited by the P1 to P9 positions. NetMHCIIpan 3.2 provides percentile binding scores to all the 9-mer sequences present in the submitted sequences. To define the HLA class II binding cores, we selected a low stringent percentile threshold of 20% in order to avoid missing potential binding cores. Peptide sequences in the HCDR2 and HCDR3 regions were predicted to bind to HLA class II molecules mainly by respectively 2 and 3 binding cores, which are shared by multiple HLA class II alleles (**Figure 1A** and **Supplementary Figures S1**, **S2**). We then evaluated the contribution of each residue of the HCDR2 and HCDR3 regions to the binding to HLA class II molecules by applying the prediction algorithm to sequences containing all possible single mutations at all the positions of the two regions. For each single mutant sequence, the number of binding cores across 46 selected HLA class II alleles was calculated. The mean effect of single mutations across the 46 HLA class II alleles was reported in an HLA class II binding heatmap as increase (red) or decrease (blue) in the number of binding cores relative to the adalimumab sequence (**Figure 1B**). Substitutions of hydrophobic positions by a hydrophilic amino acid or a proline residue tended to reduce the predicted immunogenicity scores of most of the positions in both regions. Thus, the introduction of non-polar or negatively charged amino acids seems to have a marked effect in decreasing the scores for hydrophobic amino acids, in particular at positions 47, 97 and 98, occupied by tryptophan, tyrosine and lysine residues, respectively. In contrast, introduction of hydrophobic amino acids had an opposite effect. Many substitutions are expected to lead to decreased binding of corresponding peptides to HLA class II molecules.

**Figure 2:**
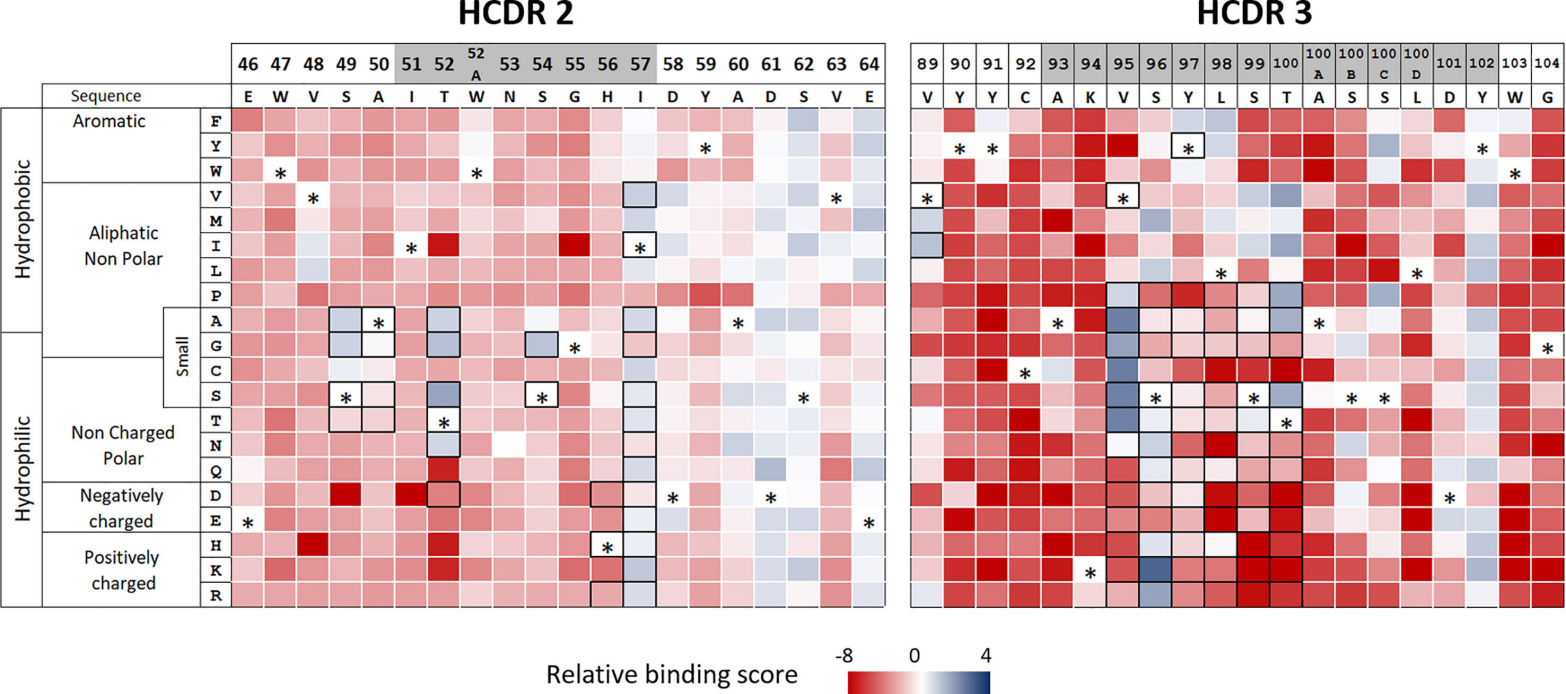
Functional analysis of HCDR2 and HCDR3 by deep mutational scanning. Libraries of monosubstituted mutants of CDR2 (left panel) and CDR3 (right panel) were screened for TNFα binding by flow cytometry. Heatmaps were established with each enrichment score of active single mutants. Enrichment score is a base 2 log function of the enrichment between sorted and unsorted yeast clones, characterized by a given amino acid substitution. Native amino acids at each position for which a score cannot be determined are represented by an asterisk. Substitutions selected to design the combinatorial libraries on each CDR are represented by black frames. CDRs are shown in grey.

Many substitutions in adalimumab HCDR2 and HCDR3 lead to a dramatic loss of binding to TNFα We also guided the mutational strategy by identifying in the HCDR2 and HCDR3 regions permissive substitutions, which neither disrupt the overall structure of the VH domain nor alter the binding to TNFα. DMS was applied by generating two libraries encompassing DNA mutations corresponding to all single amino acid changes in each HCDR region using a single NNK degenerate codon. The two libraries were cloned into a plasmid suitable for Fab expression by the yeast surface display system based on the Aga2p/Aga1p anchoring proteins. The expressed mutants were then independently sorted by flow cytometry for recognition of TNFα. For each library, plasmid DNA was sequenced for both sorted and unsorted yeasts to assess the clone frequencies in both populations. For each substitution, enrichment and relative binding score were calculated and reported as a heatmap of TNFα binding (**Figure 2**). Relative binding score varied from -8 to 2, these variations corresponding to an enrichment 256 -fold lower and 8 -fold higher, respectively, than the native sequence of adalimumab. A few positions, including T57, D61, S62 and E64 in the HCDR2 and Y102 in the HCDR3, accepted a large number of amino acid changes. Only a limited number of substitutions at positions S49, A50, T52 and S54 in the HCDR2 region and V89, V95, S96, S99, T100 and T100B in the HCDR3 region did not affect the binding to TNFα, in contrast to many other substitutions at the same positions. As a result, conservation of antibody structure and function appears as a strong bottleneck in selection of non-deleterious substitutions, while mitigation of predicted immunogenicity allows many amino acid changes.

### Screening of combinatorial libraries selected many active variants with reduced HLA class II binding scores

**Figure 3:**
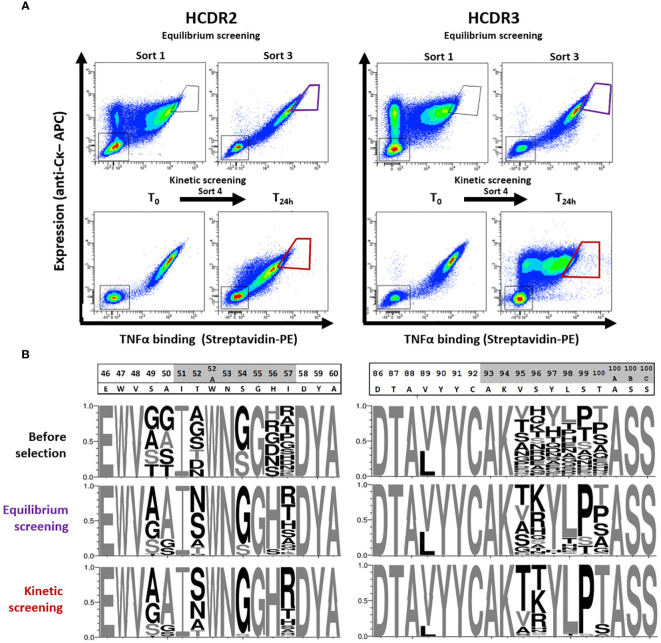
Screening process and evolution of libraries. **(A)** Libraries were screened independently by yeast surface display and FACS first by equilibrium screening using decreasing concentrations of TNFα. The first and last equilibriums selection are shown. k_off_-based kinetic screening was performed by first incubating the libraries for 3 hours with biotinylated TNFα (T_0_) followed by 24 hours of competition with unlabeled TNFα (T_24h_). At each screening step, 1% to 5% of the cells displaying the higher expression and interaction signal were sorted. **(B)** Libraries were sequenced before FACS screening and after equilibrium and kinetic screening. A random sample of 1000 sequences was used to represent each library by a weblogo. Amino acid sizes are proportional to their frequencies at the considered position and non-native amino acids are shown in black.

In order to find new active variants with reduced predicted T-cell epitope content, we designed combinatorial libraries based on the convergence of the two matrixes (**Figures 1B**, **2**). One library was designed in the HCDR2 region by introducing permissive substitutions at positions S49, A50, T52, S54, H56 and T57 (black framed **Figure 2**), which are not expected to increase the binding to HLA class II (**Figure 1B**). This library extends over 6 residues and is composed of 2496 different amino acid sequences. Another library was designed in the HCDR3 area focusing on the overlap between the three binding cores identified (in red in **Figure 1A**). As this zone is very restricted and does not contain many substitutions suitable for HLA class II binding disruption, we decided to design a combinatorial library containing all small and hydrophilic amino acids except for cysteine on six consecutive residues. We also included the possibility of a back mutation to leucine at position V89 (black framed in **Figure 2**). The main aim of the partial randomization of six consecutive residues was to take advantage of potential epistatic effects allowing substitutions that could not be identified by DMS. This library composed of 7.6 x10^6^ mutants spreads over 7 positions of the HCDR3 region and was built with a custom oligonucleotide synthesized with trinucleotide blocks. It contains all desired mutations plus the original amino acid. After cloning in a plasmid allowing their expression as Fab at the surface of yeasts, these two libraries were independently screened for TNFα binding. Due to its size, the HCDR3 library was first enriched using MACS. The screening process was composed of three rounds of sorting by flow cytometry using decreasing concentration of biotinylated TNFα and of one final round of kinetic screening, which was introduced to select mutants with a low dissociation rate (**Figure 3A**). For each sort, a diagonal gate was defined to select clones with both a high level of expression and TNFα binding. Next-generation sequencing of the HCDR2 and HCDR3 regions from transformed yeasts was performed three times i.e. before selection, after equilibrium screening and after kinetic screening (**Figure 3B**).

The distribution of amino acids in the unselected HCDR2 and HCDR3 libraries showed high diversity and amino acid frequencies consistent with the library design. At the end of the equilibrium selections, positions 50 and 56 in HCDR2 or 97 and 98 in HCDR3 showed a marked return to the parental amino acid. The other positions had rather diversified profiles with, however, a strong dominance of proline in position S99. The final selection step on dissociation rate (k_off_) produced a strong change in the profiles of some positions. Positions 89 and 100 underwent a significant enrichment towards the parental amino acid, as did 97 and 98, for which little diversity was observed already. On the contrary, positions 54 in HCDR2 and 99 in HCDR3 were highly enriched by a substituted amino acid, while other positions such as 57 or 95 still hosted multiple amino acids after screening. However, their diversity tended to reduce after the kinetic screening step. After the three selection steps, 489 and 234 variants were identified in the HCDR2 and HCDR3 regions, respectively, with 310 and 174 mutants being more enriched than adalimumab. The clones from the two libraries were then assembled in a combinatorial HCDR2+HCDR3 library, which was estimated to be approximately 1.1 x10^5^ clones in size and subjected to a new selection cycle. This combinatorial library proved to be relatively homogeneous (**Supplementary Data**). At the end of the different selections, the final sorted library kept a certain diversity, in particular at the most mutation-permissive positions. This was particularly the case for six permissive positions, four in HCDR2 (49, 52, 54 and 57, but not 50) and only two in HCDR3 (95, 96), which in the end seems very consistent with the DMS data.

**Figure 4:**
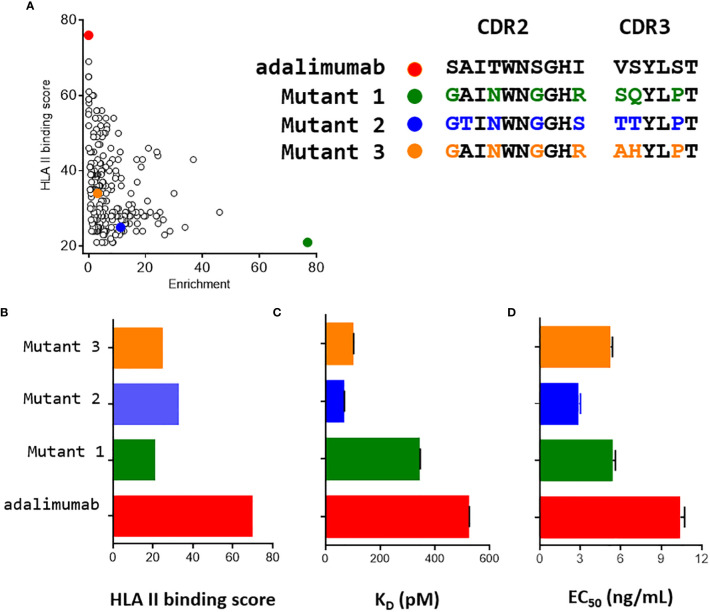
Characterization of three mutants of interest. **(A)** Two -dimensional plot of the enrichment and the HLA II binding score of the 245 mutants with an enrichment greater than that of adalimumab. Three mutants indicated in green, khaki and yellow were selected for further characterization. **(B)** Using netMHCIIpan3.2 HLA binding score were assessed separately for the HCDR2 and HCDR3 regions. These scores were plotted using the same set of colors as for A. **(C)** Affinity constants (K_D_) of the three mutants for TNFα were determined by bio layer interferometry using 6 concentrations of soluble Fab. **(D)** Neutralization activities against TNFα of the three mutants were determined using L929 murine fibroblasts and full IgG. Experiments were performed in triplicate, but in duplicate for adalimumab.

Of all unique Fab mutants identified after selection, 245 mutants shown in **Figure 4A** were found to be more enriched than adalimumab. Among those, 200 were predicted by netMHCIIpan3.2 to contain peptide sequences that would be less likely to bind well to HLA class II molecules. The vast majority of mutants incorporate substitutions within these six permissive positions, in addition to the S99P mutation. These results demonstrate that multiple sequences meet the expected requirements for selected clones for functionality and lower binding scores of corresponding peptides to HLA class II molecules.

### The Fab of selected clones exhibited a reduced HLA binding score and a better neutralization capacity than adalimumab

Three mutants were selected among the 245 identified mutants for their marked enrichment, reduced content in predicted binding of corresponding peptides to HLA II molecules and moderate sequence redundancy (**Figure 4A**). As compared to adalimumab, the three mutants showed a strong reduction of the HLA class II binding score, the number of HLA binding cores being two to three times less than for adalimumab (**Figure 4B**). All mutants expressed in Fab format had a higher affinity for TNFα than adalimumab, as assessed by Bio Layer interferometry (**Figure 4B**). The affinities of mutants 2 and 3 were respectively fifteen and four times higher than that of adalimumab, while the affinity of mutant 1 was slightly higher than that of adalimumab. For mutant 2, an increase in the affinity is clearly visible, (**Figure 4**) mainly driven by a reduction in dissociation rate (**Supplementary Figure S3**), a parameter screened for during the last round of selection.

A cell-based assay using murine L929 fibroblasts was then performed to validate the ability of these three variants expressed as IgG molecules to neutralize TNFα. The mutants were clearly active, with greater neutralizing activity than adalimumab, showing at least a 2-fold decrease in EC_50_, consistent with their greater affinity for TNFα.

## Discussion

The therapeutic efficacy of monoclonal antibodies relies mainly on their high specificity for their target and their long lifespan in the blood, but their possible immunogenicity is a major issue for their development. Repeated administration of a therapeutic mAb can promote the production of ADA, which can decrease the antibody pharmacokinetics, neutralize its therapeutic activity or trigger allergic symptoms. In this study, we aimed to reduce the predicted immunogenicity of a therapeutic antibody by identifying mutations that reduce binding of antibody peptide sequences to HLA class II molecules in positions that are permissive with respect to antibody function. These substitutions were then used to design combinatorial libraries to select variants with improved affinity for the antigen and reduced content in predicted binding peptides to HLA class II molecules. This study also shed lights on the flexibility of antibody sequences with regards to functionality and binding to HLA class II molecules.

Immunogenicity onset is very variable depending on the molecules and the pathologies. Some mAbs are known to be well tolerated while other molecules such as bococizumab and atezolizumab are described as immunogenic for a large proportion of treated patients (39). As self-proteins are generally less immunogenic than foreign proteins, humanization and generation of fully human mAbs have become a common strategy. However, even fully human antibodies may be immunogenic owing to their complementarity-determining regions (15, 40), which remain different from germinal sequences, i.e. sequences coded by the germline V and J genes from which they derive. As an example, adalimumab, a fully human anti-TNFα antibody, generates ADAs in 25 to 30% of patients suffering from rheumatoid arthritis (41). In fact, immunogenicity is in part due to immunogenic sequences (T cell epitopes) that are recognized by CD4 T lymphocytes, which provide help to B-lymphocytes to produce ADA. As CD4 T lymphocytes are counter -selected by self-peptides during their development, they preferentially recognize non-germinal sequences in the antibodies (13–16). In order to mitigate the predicted immunogenicity of mAbs, a de-immunization strategy has been proposed and consists in suppressing T cell epitopes by modifying their interactions with HLA class II molecules, thus abrogating T cell activation and ADA production (14, 22).

De-immunization examples are described in the literature for immunotoxins (21), recombinant erythropoietin (19), L-asparaginase II (20) and only a few antibodies (14, 22). In most cases, the described methods involve experimental screening of a small set of point mutants in the amino acid sequences of the therapeutic protein. To date, methods to address immunogenicity in a high -throughput manner are still lacking. In contrast, there is a very rich corpus of articles for the engineering of antibodies and the modification of other properties such as their affinity, selectivity, half-life or effector functions (42). As for most antibodies, CD4 T cell epitopes of adalimumab have been found in hypervariable loops in particular in HCDR2 and HCDR3 (15) These loops are, however, also very important for adalimumab function. HCDR3 is the most variable region of an antibody and is considered critical for its specificity (43), and HCDR2 of adalimumab is described as a key area for interaction with TNFα (44). To remove T-cell epitopes, we sought to understand how the introduction of mutations within HCDR2 and HCDR3 could decrease the prediction scores for binding to HLA class II molecules. Evaluation using an HLA-II binding algorithm seemed necessary here as a high-throughput predictor of cellular and patient responses, which unfortunately do not offer sufficient throughput to allow evaluation of hundreds or even thousands of variants. Therefore, we parallelized the evaluation of the content in potential T cell epitopes by generating virtual peptides containing every possible unique substitution and introducing their sequences in a T cell epitope prediction program. This resulted in what we termed an HLA class II binding heatmap, which summarizes in tabular form the influence of each substitution for each position on the binding to HLA class II molecules.

Peptide sequences overlapping HCDR2 and HCDR3 of adalimumab have many mutations predicted to reduce interaction with HLA-II molecules. Substitutions to hydrophilic amino acids as well as to proline generally tend to improve prediction scores at most positions. The introduction of hydrophobic amino acids on the other hand generally has the opposite effect. Several positions present substitutions likely to decrease the prediction scores, mainly hydrophobic amino acids (e.g. W47, V48, I51 in HCDR2 and V89, Y90, Y91, Y97, L98 in HCDR3), but also some polar amino acid substitutions (e.g. T52E, S49D). The best de-immunization modifications suggested by the prediction program are generally substitutions to negatively charged residues such as aspartate or glutamate or small amino acids such as glycine. Accordingly, we observe that multiple substitutions are indicated as beneficial by the prediction program, suggesting that multiple sequences should reduce the binding to HLA class II molecules and thus the predicted immunogenicity of adalimumab.

Because of the overlap between immunogenic zones and the paratope, a balance must be struck between the presence of T-cell epitope sequences and preserved functionality. Preservation of high antigenic affinity and T-cell epitope content prediction being orthogonal properties, it is likely that many sequences with reduced predicted immunogenicity are not fully functional. To determine the permissiveness of the adalimumab sequence to mutations in these two areas, we performed a DMS approach. Increasingly popular use of DMS approaches has been made possible by the coupling of high-throughput selection (e.g. display techniques) and Next-generation sequencing. This makes it possible to evaluate very quickly and systematically the impact of all possible substitutions in a protein-protein interface. The applications of DMS are very diverse, ranging from epitope mapping (45), through optimization of the affinity of monoclonal antibodies (7), to the evaluation of antigen mutations leading to immune escape, for example for viruses (46, 47). Here, we are searching for affinity-neutral mutations, similar to the concept of neutral drift in protein evolution, i.e. mutations that do not impact the folding or activity of the protein (48). Within the space of function-preserved possibilities, we are looking for variants with an orthogonal property: a lesser recognition by HLA class II molecules of the peptides they contain. This allowed us to experimentally determine the tolerance of each position of HCDR2 and HCDR3 to each possible substitution to one of the twenty proteinogenic amino acids. This exhaustive approach enables the selection of the most suitable residue(s) for each position, considering the desired properties, the affinity using DMS data and the predicted immunogenicity evaluated by bioinformatic tools.

As shown in **Figure 2**, a majority of HCDR2 and HCDR3 positions are not permissive for TNFα binding function, with negative enrichment scores except for the parent amino acid (e.g. I51, W52a, N53, G55 and H56 in HCDR2; Y90, Y91, C92, K94 and L100d in HCDR3). Other positions only tolerate a number of relatively conservative substitutions (e.g. positions 49, 50 and 54, in HCDR2; 95, 96, 99 and 100 in HCDR3), while rare positions accept a more diverse panel of amino acids (T52, I57 or Y102). As expected in a CDR, the profile of tolerated mutations is varied and clearly depends on the considered position. Overall, the DMS data indicates that the adalimumab sequence has limited permissiveness in these two CDRs, whereas the HLA class II binding heatmap offers many more possible substitutions to reduce binding of the corresponding peptides to HLA class II molecules. In this set-up, maintenance of function is probably the strongest constraint, but it is also the easiest property to screen for at high throughput. For this reason, we chose to design optimized libraries to assemble mutations combining lower potential immunogenicity with maintenance of function. At the end of the selection steps, active clones showed sequence diversity at the 6 main positions (49, 52, 54 and 57 in HCDR2, 95 and 96 in HCDR3), in good agreement with the heatmaps obtained by DMS. Singularly, the S99P mutation, which was identified as slightly unfavorable by DMS, turns out to be present in a large majority of the mutants, perhaps benefiting from compensatory effects due to the presence of additional mutations in its vicinity.

Among these 245 active sequences, nearly 200 are predicted to have a lower number of interaction cores with HLA class II molecules than adalimumab, i.e. a lower predicted immunogenicity. These alternative sequences incorporate between 2 and 9 mutations, with an average of 6.7 mutations relative to the parental sequence. The vast majority of these adalimumab mutations identified in this work are novel. Affinity maturation approaches have identified a number of mutations in the light chain and in HCDR1 mainly (2, 49). Interestingly, the only common position is position 96 in HCDR3 which is occupied by a serine residue, which was previously identified as permissive in both studies. Another study looking at the pH dependence of antibody/antigen interactions also showed that histidine could be introduced into the HCDR3 sequence, in particular at position L98 (50). The combination of steady -state screening and antigen/antibody stability screening allowed us to retain only highly active sequences, as evidenced by the affinities of the three Fab-expressed clones and their ability to neutralize TNFα in an IgG format. The stringency of the functional screen and the composition of the starting library certainly have an impact on the diversity of clones obtained *in fine*. It seems therefore possible to introduce fewer functional constraints to offer more choices of amino acids and thus potentially increased de-immunization.

In addition to immunogenicity, other properties are essential to ensure the developability of therapeutic antibodies and to minimize the risk of failure in clinical phases. Thermal stability, absence of post-translational modifications and propensity to form aggregates are also very important parameters to consider when developing monoclonal antibodies (51). Our approach could also be applied to modify the areas involved in these undesirable properties. In this case, the existence of a panel of a few tens or hundreds of variants is a great advantage, and may allow us to find the best compromise between these often independent properties.

## Data availability statement

The datasets presented in this study can be found in online repositories. The names of the repository/repositories and accession number(s) can be found here: PRJNA953338 (SRA).

## Author contributions

BM and HN designed the experiments, analyzed the data and wrote the manuscript. CS and RS designed and performed the experiments, analyzed the data and corrected the manuscript. SD, WQ, YL, AL, AM and EC designed and performed the experiments and analyzed the data. All authors contributed to the article and approved the submitted version

## Aknowledgments

The authors would like to acknowledge Roselaine Campos Targino for carrying out the TNFalpha neutralization experiments.
