# Supplementary material for: Combining deep mutational scanning to heatmap of HLA class II binding of immunogenic sequences to preserve functionality and mitigate predicted immunogenicity
Source: Front Immunol. 2023 Jul 28;14:1197919. doi: 10.3389/fimmu.2023.1197919 (PMC10416631; doi:10.3389/fimmu.2023.1197919)
Supplement: Supplementary file 1 [file DataSheet_1.pdf]

CDRH2 (positions 51–57)

Alleles	46	47	48	49	50	51	52	52A	53	54	55	56	57	58	59	60	61	62	63	64
	E	W	V	S	A	I	T	W	N	S	G	H	I	D	Y	A	D	S	V	E
DRB1_0101	100	17	100	100	100	38	100	85	100	100	100	100	70	100	100	85	100	100	100	100
DRB1_0301	100	75	100	100	100	60	100	100	100	100	80	100	100	30	100	100	75	100	100	100
DRB1_0302	100	32	100	100	100	25	100	100	100	100	65	100	100	31	85	100	95	100	100	100
DRB1_0401	100	5	100	100	100	28	100	60	100	100	100	100	43	100	100	100	100	100	95	100
DRB1_0402	100	32	100	100	100	42	100	85	100	100	100	100	80	100	100	100	100	100	90	100
DRB1_0403	100	14	100	100	100	40	100	85	100	100	100	100	65	100	100	100	100	100	80	100
DRB1_0404	100	15	100	100	100	44	100	90	100	100	100	100	65	100	100	100	100	100	85	100
DRB1_0405	100	8	100	100	100	65	100	70	100	100	100	100	60	100	90	100	100	100	100	80
DRB1_0407	100	5	100	100	100	23	100	70	100	100	100	100	49	100	100	100	100	100	95	100
DRB1_0411	100	22	100	100	100	49	100	90	100	100	100	100	75	100	100	100	100	100	85	100
DRB1_0701	100	14	100	100	100	27	100	80	100	100	100	100	70	100	75	85	100	100	100	100
DRB1_0802	100	11	33	100	100	65	100	95	100	100	100	100	80	100	100	100	85	100	90	100
DRB1_0901	100	9	100	100	100	21	100	47	100	100	100	100	65	100	100	70	100	100	100	100
DRB1_1101	100	29	100	100	100	50	100	90	100	100	100	100	85	100	100	100	85	100	100	100
DRB1_1102	100	48	100	100	100	37	100	100	100	100	100	100	85	100	100	100	85	100	80	100
DRB1_1103	100	43	100	100	100	55	100	90	100	100	100	100	85	100	100	100	100	100	75	100
DRB1_1104	100	37	100	100	100	55	100	90	100	100	100	100	85	100	100	100	85	100	80	100
DRB1_1201	100	55	100	100	100	55	100	100	100	100	100	100	85	100	100	100	85	100	90	100
DRB1_1301	100	48	100	100	100	37	100	100	100	100	100	100	85	100	100	100	85	100	80	100
DRB1_1302	100	37	100	100	100	7	100	100	100	100	90	100	75	100	85	100	95	100	100	100
DRB1_1303	100	40	100	100	100	21	100	100	100	100	100	100	70	100	80	100	90	100	100	100
DRB1_1304	100	55	100	100	100	42	100	100	100	100	100	100	85	100	100	100	85	100	80	100
DRB1_1401	100	38	100	100	100	42	100	90	100	100	100	100	75	100	100	100	85	100	85	100
DRB1_1402	100	20	100	100	100	21	100	100	100	100	100	100	65	80	100	100	95	100	100	100
DRB1_1501	100	36	100	100	100	34	100	85	100	100	100	100	80	100	100	100	85	100	90	100
DRB1_1601	100	18	100	100	65	100	100	70	100	100	100	100	80	100	75	85	100	100	100	100
DRB3_0101	100	43	100	100	100	33	100	100	100	100	26	100	100	19	70	100	90	100	100	100
DRB3_0202	100	11	100	100	100	9	100	85	100	100	100	100	80	100	90	100	95	100	100	100
DRB3_0301	100	33	100	100	100	19	100	100	100	100	100	100	80	100	100	100	90	100	95	100
DRB4_0101	100	45	100	100	100	55	100	90	100	100	100	100	85	100	100	100	90	100	90	100
DRB5_0101	100	30	100	100	100	55	100	90	100	100	100	100	80	100	75	100	100	100	80	100
DRB5_0102	100	25	100	100	100	46	100	90	100	100	100	100	75	100	75	100	100	100	85	100
HLA-DPA10103-DPB10201	100	39	100	100	100	55	100	80	100	100	100	100	80	100	70	100	80	100	100	85
HLA-DPA10103-DPB10401	100	38	100	100	55	75	100	85	100	100	100	100	80	100	75	100	85	100	100	85
HLA-DPA10103-DPB10402	100	32	100	100	100	70	100	85	100	100	100	100	80	100	70	100	80	100	100	100
HLA-DPA10201-DPB10101	100	47	100	100	100	65	100	85	100	100	100	100	80	100	75	100	80	100	100	85
HLA-DPA10201-DPB11401	100	25	100	100	100	85	100	95	100	100	100	100	80	100	100	85	85	100	100	100
HLA-DPA10202-DPB10501	100	60	100	100	100	75	100	95	100	100	100	100	80	100	80	100	80	100	80	100
HLA-DQA10101-DQB10501	100	43	100	100	100	28	100	23	25	100	100	32	65	100	80	100	100	100	100	70
HLA-DQA10102-DQB10502	100	43	100	100	100	29	100	100	19	100	100	31	44	80	100	100	100	100	100	55
HLA-DQA10102-DQB10602	100	10	24	100	100	35	100	36	30	100	100	100	55	100	100	100	100	70	100	100
HLA-DQA10201-DQB10201	100	28	100	100	100	100	100	29	100	100	24	100	22	55	100	100	100	100	100	70
HLA-DQA10301-DQB10302	100	17	100	100	100	100	100	22	13	100	100	100	14	100	100	100	100	100	100	55
HLA-DQA10401-DQB10402	100	17	100	100	100	100	100	26	18	100	100	100	19	100	100	60	100	100	100	60
HLA-DQA10501-DQB10201	100	36	100	100	100	100	100	21	100	100	100	100	16	45	65	100	100	100	100	75
HLA-DQA10501-DQB10301	100	11	100	100	100	100	100	11	100	100	100	100	33	100	100	100	100	32	100	100
Binding cores	0	15	0	0	0	3	0	1	3	0	0	0	3	1	0	0	0	0	0	0

Figure S1: percentile binding scores of the region encompassing the a adalimumab HCDR2

The sequence was submitted to netMHCIIpan3.2 using a panel of preponderant HLA class II alleles. Percentile ranks were reported in the first position of the considered 9-mer and were generated by comparison with five million random peptides selected from SWISSPROT database. A 9-mer sequence was considered as a HLA binding core for a percentile rank below 20% (red). Poor binding correspond to percentile ranks over 20 (blue to white).

									HCDR3											
	89	90	91	92	93	94	95	96	97	98	99	100	100A	100B	100C	100D	101	102	103	104
Alleles	V	Y	Y	C	A	K	V	S	Y	L	S	T	A	S	S	L	D	Y	W	G
DRB1_0101	100	5	100	100	100	100	3	100	4	50	100	100	100	100	100	100	85	39	100	100
DRB1_0301	39	70	100	100	100	100	80	100	100	55	100	100	100	90	100	100	100	95	100	100
DRB1_0302	70	23	100	100	100	100	45	100	34	65	100	100	100	95	100	100	80	100	80	100
DRB1_0401	100	6	100	100	100	100	0	100	0	23	100	100	100	100	100	100	95	43	100	100
DRB1_0402	100	19	18	100	100	100	7	100	100	27	100	100	100	100	100	95	100	80	100	100
DRB1_0403	100	18	100	100	100	100	1	100	10	24	100	100	95	100	100	100	100	75	100	100
DRB1_0404	100	21	18	100	100	100	1	100	10	26	100	100	95	100	100	100	100	75	100	100
DRB1_0405	100	9	100	100	100	100	1	100	0	20	100	100	100	100	100	100	90	55	100	100
DRB1_0407	100	9	100	100	100	100	3	100	1	29	100	100	100	100	100	100	95	55	100	100
DRB1_0411	100	19	100	100	100	100	2	100	12	24	100	100	100	100	100	95	100	80	100	100
DRB1_0701	100	5	100	100	100	100	100	5	3	13	100	100	100	100	100	100	47	100	100	100
DRB1_0802	70	14	14	100	100	100	4	100	17	70	100	100	100	100	100	95	100	80	100	100
DRB1_0901	100	2	100	100	100	100	3	100	1	27	100	100	100	100	100	100	35	30	100	100
DRB1_1101	19	100	12	100	100	100	25	100	44	80	100	100	95	100	100	95	100	75	100	100
DRB1_1102	31	100	41	100	100	100	39	100	100	44	100	100	100	100	100	95	90	90	100	100
DRB1_1103	19	100	25	100	100	100	27	100	100	55	100	100	90	100	100	85	100	80	100	100
DRB1_1104	15	100	20	100	100	100	21	100	100	55	100	100	90	100	100	85	100	75	100	100
DRB1_1201	33	100	100	100	100	100	27	100	100	21	100	100	100	100	100	80	100	100	100	100
DRB1_1301	31	100	41	100	100	100	39	100	100	44	100	100	100	100	100	95	90	90	100	100
DRB1_1302	80	40	100	100	100	100	100	27	100	32	100	100	100	100	100	100	80	100	100	100
DRB1_1303	65	18	100	100	100	100	29	100	19	50	100	100	100	100	100	100	85	80	100	100
DRB1_1304	30	100	44	100	100	100	45	100	100	55	100	100	100	100	100	85	100	100	100	100
DRB1_1401	47	28	100	100	100	100	17	100	100	21	100	100	100	100	100	95	100	85	100	100
DRB1_1402	80	19	100	100	100	100	15	100	13	44	100	100	100	100	100	100	95	80	100	100
DRB1_1501	100	100	15	100	100	100	14	100	100	43	100	100	100	100	100	65	100	100	100	100
DRB1_1601	100	9	3	100	100	100	13	100	13	65	100	100	85	100	100	85	100	55	100	100
DRB3_0101	50	18	100	100	100	100	100	40	24	34	100	100	100	75	100	100	100	60	100	100
DRB3_0202	80	14	100	100	100	100	100	20	10	100	100	100	100	95	100	100	85	60	100	100
DRB3_0301	75	32	100	100	100	100	28	20	100	32	100	100	100	100	100	100	85	100	80	100
DRB4_0101	47	100	100	100	100	100	28	100	100	55	100	100	100	100	100	90	100	90	100	100
DRB5_0101	60	11	14	100	100	100	22	100	25	55	100	100	95	100	100	95	100	75	100	100
DRB5_0102	60	7	10	100	100	100	12	100	20	70	100	100	95	100	100	95	100	70	100	100
HLA-DPA10103-DPB10201	100	27	100	12	100	100	100	100	28	39	100	100	70	100	100	47	100	100	49	100
HLA-DPA10103-DPB10401	55	27	100	12	100	100	100	100	29	48	100	100	70	100	100	47	100	100	49	100
HLA-DPA10103-DPB10402	55	12	100	7	100	100	100	100	22	46	100	100	75	100	100	60	100	55	60	100
HLA-DPA10201-DPB10101	60	32	100	13	100	100	100	100	27	38	100	100	80	100	100	60	100	100	60	100
HLA-DPA10201-DPB11401	100	11	100	100	100	100	4	100	17	65	100	100	100	100	100	100	95	70	100	100
HLA-DPA10202-DPB10501	19	100	100	13	100	100	40	100	46	55	100	100	90	100	100	80	100	80	100	100
HLA-DQA10101-DQB10501	100	48	100	100	100	55	100	100	100	42	100	100	100	100	100	37	100	100	100	100
HLA-DQA10102-DQB10502	100	46	100	100	100	55	100	100	100	39	100	100	100	100	100	55	100	100	60	100
HLA-DQA10102-DQB10602	100	33	100	100	11	100	100	9	100	12	100	41	100	100	100	100	100	70	60	13
HLA-DQA10201-DQB10201	100	40	100	100	100	100	47	32	20	6	47	100	100	100	100	100	65	100	49	100
HLA-DQA10301-DQB10302	100	44	100	100	100	100	49	33	20	7	100	46	100	100	100	100	55	35	100	23
HLA-DQA10401-DQB10402	100	31	100	100	100	100	46	29	17	6	100	100	100	100	100	100	60	60	41	28
HLA-DQA10501-DQB10201	100	38	100	100	100	100	100	46	30	11	55	100	100	100	100	100	65	60	44	100
HLA-DQA10501-DQB10301	100	13	100	100	13	100	100	10	100	19	100	41	100	100	100	100	42	32	14	5
Binding cores	4	20	8	5	2	0	16	3	16	7	0	0	0	0	0	0	0	0	1	2

Figure S2: percentile binding scores of the region encompassing the a adalimumab HCDR3
Same legend as figure S1

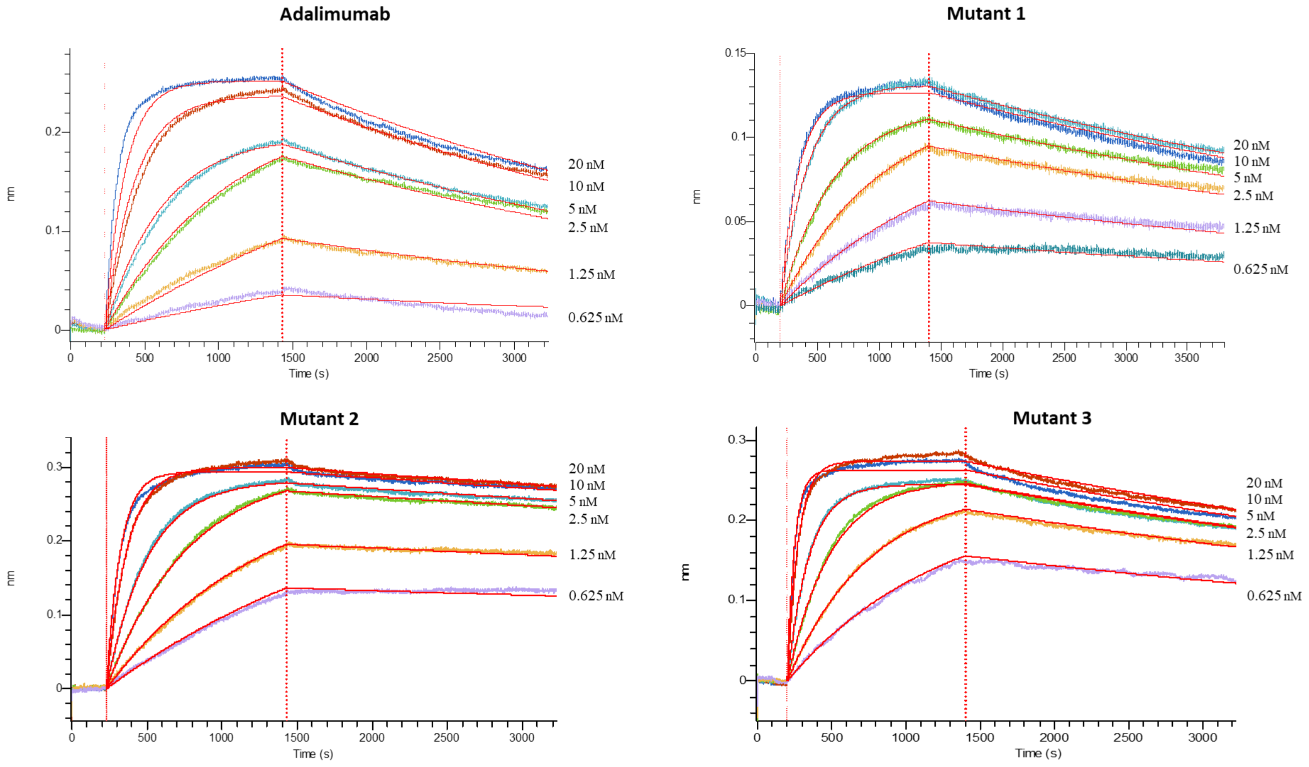

	CDRH2	CDRH3	k_a (.10^4 1/Ms)	k_d (.10^{-6} 1/s)	K_D (pM)
**Adalimumab**	SAITWNSGHI	VSYLST	39,8 (± 0,05)	170 (± 0,18)	428
**Mutant 1**	GAINWNGGHR	SQYLPT	43,9 (± 0,03)	152 (± 0,1)	345
**Mutant 2**	GTINWNGGHS	TTYLPT	64,0 (± 0,07)	16 (± 0,1)	26
**Mutant 3**	GAINWNGGHR	AHYLPT	134 (± 0,01)	136 (± 0,10)	102

Figure S3:
Affinity of selected Fab molecules for human TNFα determined by bio-layer interferometry. BLI sensorgrams showing the binding of Adalimumab Fab and Fab molecules corresponding to mutants 1,2 and 3 to biotinylated human TNFα (top panel) immobilized on Streptavidin (SA) biosensor tips. Data are shown as colored lines at different concentrations of Fab and red lines are the best fit of the data.

>VH_Adalimumab_(CodonOptimized)

GAAGTGCAGCTGGTAGAGAGCGGAGGTGGACTGGTTCAGCCTGGTCGCTCT
TTGCGTCTGAGTTGTGCAGCTTCTGGGTTCACCTTTGACGACTATGCCATGCA
TTGGGTCAGACAGGCTCCCGGAAAAGGGCTTGAGTGGGTTTCCGCCATAAC
GTGGAATTCAGGCCACATCGACTATGCCGATAGCGTGGAAGGGAGGTTCACC
ATTTCCCGGGATAATGCGAAGAACAGCCTGTATCTGCAGATGAACAGCCTTCG
AGCTGAGGATACAGCCGTGTACTACTGCGCAAAGGTGTCCTACCTCTCAACTG
CATCCTCTCTCGACTACTGGGGCCAAGGCACACTGGTCACTGTGAGTAGC

>VL_Adalimumab_(CodonOptimized)

GACATCCAGATGACACAGTCTCCATCCAGCCTTAGCGCATCTGTTGGCGATAG
AGTGACCATAACCTGTCGAGCCTCTCAAGGCATTCGCAACTATCTGGCCTGGT
ATCAGCAGAAACCGGGAAAAGCGCCTAAGCTCCTGATCTATGCTGCCTCAAC
TCTGCAGTCAGGGGTACCTAGCCGGTTCAGTGGCAGTGGATCCGGTACTGAC
TTCACACTGACGATTAGCTCCTTGCAACCCGAAGATGTCGCTACCTACTACTG
CCAGAGGTACAATCGTGCACCCTACACCTTTGGTCAGGGGACAAAGGTGGA
GATCAAG
